# Functional MRI-Specific Alterations in Executive Control Network in Mild Cognitive Impairment: An ALE Meta-Analysis

**DOI:** 10.3389/fnagi.2020.578863

**Published:** 2020-10-09

**Authors:** Wenwen Xu, Shanshan Chen, Chen Xue, Guanjie Hu, Wenying Ma, Wenzhang Qi, Xingjian Lin, Jiu Chen

**Affiliations:** ^1^Department of Neurology, The Affiliated Brain Hospital of Nanjing Medical University, Nanjing, China; ^2^Department of Radiology, The Affiliated Brain Hospital of Nanjing Medical University, Nanjing, China; ^3^Institute of Brain Functional Imaging, Nanjing Medical University, Nanjing, China; ^4^Institute of Neuropsychiatry, The Affiliated Brain Hospital of Nanjing Medical University, Nanjing, China

**Keywords:** mild cognitive impairment, resting state, executive control network, the amplitude of low frequency fluctuation, regional homogeneity, functional connectivity

## Abstract

**Background:** Mild cognitive impairment (MCI) is regarded as a transitional stage between normal aging and Alzheimer's disease (AD) dementia. MCI individuals with deficits in executive function are at higher risk for progressing to AD dementia. Currently, there is no consistent result for alterations in the executive control network (ECN) in MCI, which makes early prediction of AD conversion difficult. The aim of the study was to find functional MRI-specific alterations in ECN in MCI patients by expounding on the convergence of brain regions with functional abnormalities in ECN.

**Methods:** We searched PubMed, Embase, and Web of Science to identify neuroimaging studies using methods including the amplitude of low frequency fluctuation/fractional amplitude of low-frequency fluctuation, regional homogeneity, and functional connectivity in MCI patients. Based on the Activation Likelihood Estimation algorithm, the coordinate-based meta-analysis and functional meta-analytic connectivity modeling were conducted.

**Results:** A total of 25 functional imaging studies with MCI patients were included in a quantitative meta-analysis. By summarizing the included articles, we obtained specific brain region changes, mainly including precuneus, cuneus, lingual gyrus, middle frontal gyrus, posterior cingulate cortex, and cerebellum posterior lobe, in the ECN based on these three methods. The specific abnormal brain regions indicated that there were interactions between the ECN and other networks.

**Conclusions:** This study confirms functional imaging specific abnormal markers in ECN and its interaction with other networks in MCI. It provides novel targets and pathways for individualized and precise interventions to delay the progression of MCI to AD.

## Introduction

Mild cognitive impairment (MCI) is regarded as a transitional stage between normal aging and Alzheimer's disease (AD) dementia (Jia et al., 2015). Executive function is an important high-level cognitive process and a function of flexible integration and cooperative operation of different cognitive processes to achieve a specific goal (Trossman et al., 2020). The executive function of MCI patients is significantly impaired, which is mainly reflected in planning ability, continuous attention, and other aspects (Trossman et al., 2020). Hence, executive function impairment is associated with severe and lasting progression to AD in MCI individuals compared to other cognitive impairment. As is well-known, the executive control network (ECN) in MCI, which is a wide range of brain regions responsible for executive function shows different changes in brain regions in different studies (Joshi et al., 2019). There is no doubt that studying the specific abnormal biomarkers of executive function is a necessity due to a lack of consistent results.

Resting state functional MRI (rsfMRI) has superiority in detecting several neuropsychiatric disorders in which the changes in blood oxygen level signal intensity are considered an indirect tool associated with function in specific regions of the human brain (Wee et al., 2016). Current studies usually evaluate functional alterations in the ECN by the following methods in rsfMRI: (1) the amplitude of low-frequency fluctuation (ALFF)/fractional amplitude of low-frequency fluctuation (fALFF), (2) regional homogeneity (ReHo), and (3) functional connectivity (FC). The ALFF and fALFF are methods that measure the amplitude of spontaneous regional brain activity by calculating the square root of the power spectrum in the low-frequency range. The fALFF method has been confirmed to be more sensitive compared with ALFF (Yang et al., 2019). Numerous studies indicate that the specific patterns of ALFF and fALFF provide insights into the mechanism in AD and MCI patients (Yang et al., 2018). ReHo is widely used in the study of various mental disorders and has been demonstrated to have a high test–retest reliability in the study of the consistency of brain activity. A large amount of evidence shows a correlation between the ReHo value of abnormal brain areas and clinical symptoms (Luo et al., 2018). FC usually reflects the connectivity between brain regions to reveal whether there is connectivity disruption or compensation based on independent component analysis and seed point (Liu et al., 2020). All three methods have their own advantages in studying ECN in patients with MCI, but the results are unanimous.

ECN, mainly anchored in the bilateral dorsolateral prefrontal lobe, ventral prefrontal lobe, frontal insular cortex, and parietal lobe, has extensive connections with other regions (Wu et al., 2014). ECN plays an important role in the integration of sensory and memory information, the regulation of cognition and behavior, and the working memory process with the prefrontal lobe as its core (Petersen et al., 2019). A combined arterial spin labeling (ASL), perfusion, and rsfMRI study confirms that the brain regions in AD where cerebral blood flow and ALFF decrease predict a disruption in the ECN (Zheng et al., 2019). However, the lack of consistent results for specific alterations in ECN fail to provide specific imaging markers in predicting the transition from MCI to AD. Therefore, to explore the specific imaging markers in ECN is essential to determine if these early alterations will serve as sensitive predictors of clinical decline and, eventually, as markers of MCI progress to AD by summarizing previous studies.

Currently, the widely recognized resting state brain networks include the default mode network (DMN), ECN, salience network (SAL), dorsal attention network (DAN), fronto-parietal network (FPN), and sensory-motor network (SMN, a combination of three originally separate RSNs that correspond to the primary auditory, primary visual, and somatomotor cortices) (Chen et al., 2016). The components of each network are partially overlapped with other networks, forming dynamic interactions, which is considered to be of great significance in behavioral function (Brier et al., 2012). There has been evidence of possible interactions between the ECN and other networks (Yuan B. et al., 2016). Cognitive decline or disease would result from the destruction of certain aspects of this connectivity network (Leech and Sharp, 2014). As a result, it is necessary to investigate the mechanisms underlying the interaction between the known networks in MCI patients.

Anatomical/activation likelihood estimation (ALE) is an effective coordinate-based meta-analysis (CBMA), which avoids laboratory bias in quantifying consistent imaging findings across studies (Robinson et al., 2012). ALE has been used extensively in rsfMRI studies, and it is suggested that ALE may provide image-specific markers (Robinson et al., 2010). In total, each individual coordinate from each study that is presented as a 3-D Gaussian probability distribution. ALE maps were produced by combining the distributions from all the studies in which significance was determined by using the threshold of *p* < 0.05 (Doucet et al., 2020). This method was applied in the quantification of the location and extent of cerebellar changes across the main frontotemporal dementia and neuroimaging in Parkinson's disease dementia (Chen et al., 2019; Weil et al., 2019). Because it has been used extensively in degenerative diseases, it is reasonable to use it in MCI patients. To our knowledge, so far, it is rare that a meta-analysis is made on the functional specific alterations of the ECN in MCI patients. As a result, a quantitative meta-analysis of published neuroimaging studies using the ALE algorithm were conducted to provide an overview of the current literature on specific alterations of the ECN.

Hence, the aim of the present study was to evaluate the specific functional alterations in the ECN more comprehensively and discuss the functional imaging-specific abnormal markers in ECN and its interaction with other networks in MCI by using three techniques. It was hypothesized that, in MCI, (1) the three indexes of the ECN would show the special imaging abnormal markers, and (2) these specific brain regions would exist abnormal interactions with other networks.

## Methods

### Literature Search and Study Selection

The meta-analysis of neuroimaging studies was conducted according to the PRISMA statement and recorded using the suggested checklist.

#### Search Strategy

PubMed, Embase, and Web of science were thoroughly and systematically searched. Search keywords were as follows: (1) (“functional magnetic resonance imaging” [MeSH] OR “RESTING STATE” [MeSH]) AND [“mild cognitive impairment” [MeSH] AND “Executive Control” [MeSH] AND “Functional connectivity”]; (2) (“functional magnetic resonance imaging” [MeSH] OR “RESTING STATE” [MeSH]) AND (“mild cognitive impairment” [MeSH] AND “regional homogeneity”); and (3) (“functional magnetic resonance imaging” [MeSH] OR “RESTING STATE” [MeSH]) AND “mild cognitive impairment” [MeSH] AND (“fractional amplitude of low frequency fluctuation” OR “amplitude of low frequency fluctuation”) (Supplementary Table 1).

**Figure 1:**
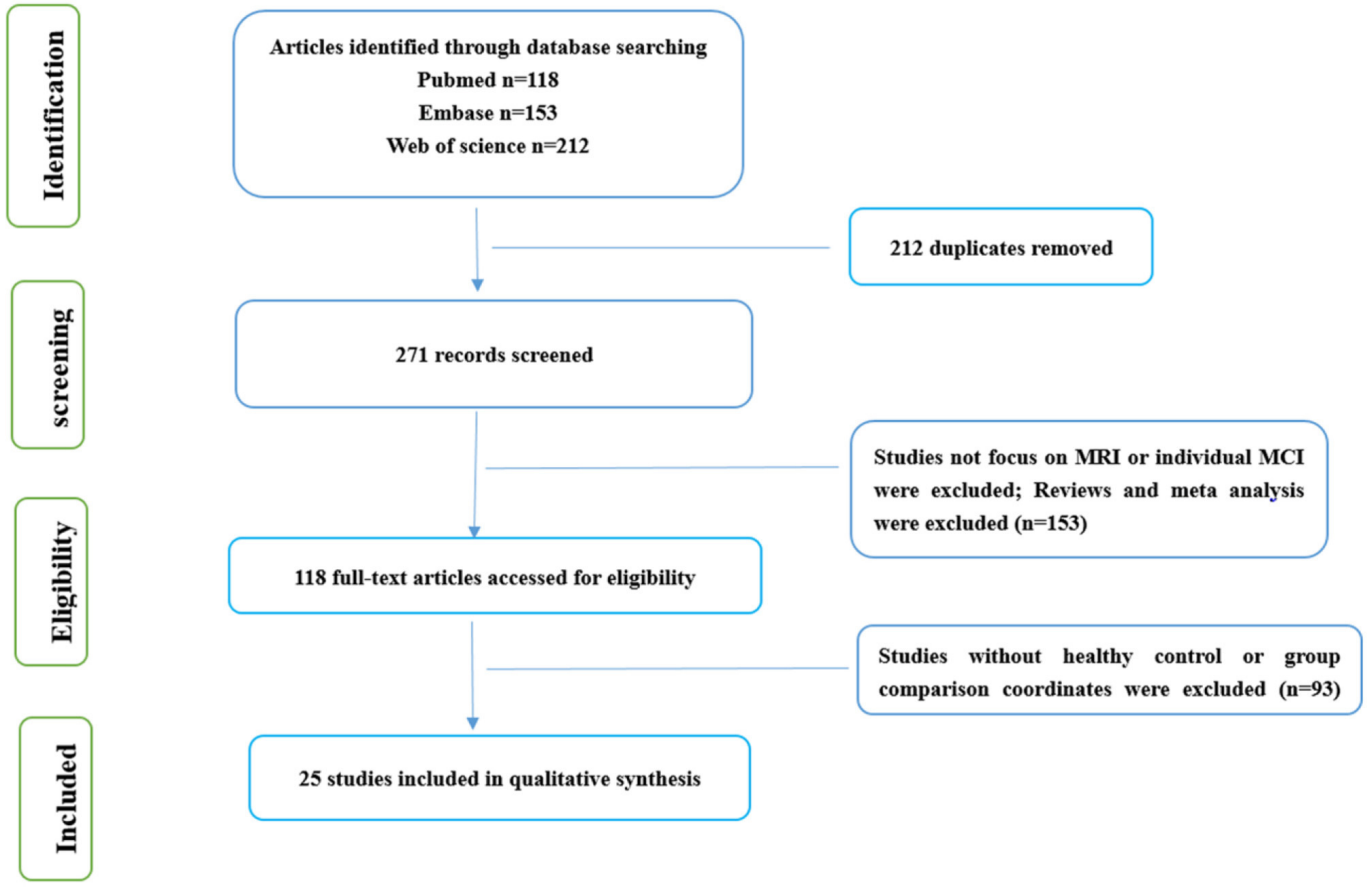
Flow of information through the different phases of a systematic review.

Only publications in English were selected. A total of 271 specific articles were screened. The flowchart of the literature search and selection strategy is shown in Figure 1.

#### Inclusion and Exclusion Criteria

All articles related to rsfMRI investigations on MCI were included in our study. To make comparisons between healthy controls (HCs) and MCI, information about Talairach or the Montreal Neurologic Institute (MNI) were required in these investigations.

If articles were based on other diseases, such as schizophrenia, depression, et al. they were eliminated. We dropped secondary processing of literature, such as reviews and meta-analysis articles. Case studies without group-level statistics were excluded. Articles whose necessary data could not be obtained even after contacting the author/s were excluded. Articles based on region of interest analysis were excluded.

#### Data Extraction and Quality Assessment

Two researchers conducted selection, data extraction, and cross-checks independently. A third reviewer participated in judgment when disagreements appeared of any kind. First, we selected articles related to ECN. Second, articles with respect to the abnormal functional network in the use of ALFF/fALFF and ReHo were included. All the abnormal brain regions extracted from the articles were based on brain regions in the ECN included in the articles.

### Data Analysis Procedures

Comparing MCI with HCs, we separated the three different methods by decreasing and increasing and then calculated them on the software: increased ALFF/ fALFF (*n* = 251; 19 foci); decreased ALFF/fALFF (*n* = 414; 36 foci); increased ReHo (*n* = 222; 17 foci); decreased ReHo (*n* = 202; 18 foci); increased FC (*n* = 136; 19 foci); decreased FC (*n* = 76; 17 foci).

An ALE meta-analysis was conducted with a Java-based version of Ginger ALE 2.3.6 (http://www.brainmap.org/ale) (Eickhoff et al., 2012). The aim of ALE was to assess the convergence of the difference between MCI and HC groups in terms of foci across studies. First, we used a text file to read foci data that are imported into the software (Eickhoff et al., 2012). A threshold at *p* < 0.05 using the false discovery rate (FDR) was set in ALE maps (Eickhoff et al., 2012). Last, the maps were covered into the MNI 152 template and viewed with Dpabi software (http://fmri.org/dpabi).

## Results

### Search Results

The study characteristics and results are summarized in Table 1.

**Table 1:** Demographic characteristics of the included studies.

**Study**	**Imaging modality**	***N***	**Age (SD)**	**Gender (male/female)**	**MMSE (SD)**	**Group contrasts**	**Foci**	**Correction for multiple comparisons**
**ALFF**								
Zhuang et al. (2012)	rs-fMRI	MCI 47 HC 33	71.957 (4.77) 72.848 (3.39)	28/19 18/15	26.979 (1.525) 28.182 (1.334)	MCI>HC MCI < HC	1 0	*P < * 0.05 (cor)
Yin et al. (2014)	rs-fMRI	MCI 22 HC 11	62.1 (8.1) 66.6 (8.7)	12/10 2/9	24.6 (3.2) 29.2 (1.1)	MCI>HC MCI < HC	1 4	*P < * 0.05 (cor)
Zhao et al. (2014)	rs-fMRI	MCI 20 HC 18	65.11 (9.92) 66.81 (7.43)	8/12 8/10	25.21 (2.24) 29.31 (1.22)	MCI>HC MCI < HC	4 3	*P < * 0.01 (cor)
Wang et al. (2011)	rs-fMRI	MCI 16 HC 22	69.38 (7.00) 66.55 (7.67)	7/9 7/15	26.50 (1.03) 28.59 (0.59)	MCI>HC MCI < HC	1 4	*P < * 0.05 (cor)
Liu X. et al. (2014)	rs-fMRI	MCI 46 HC 32	71.89 (4.88) 72.84 (3.46)	27/19 18/14	–	MCI>HC MCI < HC	1 1	*P < * 0.05 (cor)
Zhuang et al. (2019)	rs-fMRI	MCI 35 HC 26	71.06 (4.42) 69.10 (5.31)	23/12 16/10	27.30 (1.46) 28.14 (1.36)	MCI>HC MCI < HC	1 1	*P < * 0.05 (cor)
Cai et al. (2017)	rs-fMRI	MCI 39 HC 38	72.4 (5.01) 73.92 (3.90)	19/20 19/19	25.51 (2.88) 29.28 (0.88)	MCI>HC MCI < HC	5 8	*P < * 0.05 (cor)
Jia et al. (2015)	rs-fMRI	MCI 8 HC 15	74.1 (7.8) 70.2 (7.1)	2/6 8/7	27.0 (2.3) 29.2 (1.3)	MCI>HC MCI < HC	0 1	*P < * 0.01 (cor)
Yang et al. (2018)	rs-fMRI	aMCI 55 HC 57	67.51 (9.62) 63.77 (8.09)	27/28 22/35	24.66 (4.20) 28.14 (2.13)	MCI>HC MCI < HC	0 3	*P < * 0.05 (cor)
Xi et al. (2013)	rs-fMRI	MCI 18 HC 20	67.39 (7.67) 65.42 (5.75)	8/10 9/9	25.16 (3.43) 28.14 (1.84)	MCI>HC MCI < HC	1 2	*P < * 0.05 (cor)
**fALFF**								
Liu et al. (2013)	rs-fMRI	MCI 38 HC 34	74.91 (5.88) 77.30 (7.33)	16/16 15/13	–	MCI>HC MCI < HC	2 1	*P < * 0.05 (cor)
Yang et al. (2019)	rs-fMRI	MCI 52 HC 55	68.06 (9.32) 63.41 (7.97)	26/26 22/23	24.52 (4.27) 28.07 (2.14)	MCI>HC MCI < HC	0 2	*P < * 0.05 (cor)
Yang et al. (2018)	rs-fMRI	MCI 55 HC 57	67.51 (9.62) 63.77 (8.09)	27/28 22/35	24.66 (4.20) 28.14 (2.13)	MCI>HC MCI < HC	0 3	*P < * 0.05 (cor)
Zhou et al. (2020)	rs-fMRI	aMCI 24 HC 32	69.8 (6.2) 67.9 (6.4)	10/14 14/18	23.9 (3.6) 28.0 (1.9)	MCI>HC MCI < HC	2 0	*P < * 0.001 (cor)
	rs-fMRI	aMCI 47 HC 32	69.1 (6.5) 67.2 (6.6)	23/24 14/18	23.3 (3.7) 28.4 (1.8)	MCI>HC MCI < HC	0 1	*P < * 0.05 (cor)
**ReHo**								
Liu Z. et al. (2014)	rs-fMRI	MCI 12 HC 12	59.30 (3.3) 60.6 (5.8)	1/11 4/8	26.4 (0.9) 29.8 (0.4)	MCI>HC MCI < HC	3 4	*P < * 0.01 (cor)
Min et al. (2019)	rs-fMRI	MCI 10 HC 10	69.80 (2.658) 69.90 (2.601)	5/5 5/5	25.90 (0.738) 29.30 (0.823)	MCI>HC MCI < HC	3 4	*P < * 0.05 (uncor)
Cai et al. (2018)	rs-fMRI	MCI 50 HC 53	72.3 (6.86) 76.08 (6.45)	24/26 29/24	24.3 (2.45) 28.2 (2.13)	MCI>HC MCI < HC	1 1	*P < * 0.01 (cor)
	rs-fMRI	MCI 20 HC 53	69.76 (6.48) 76.08 (6.45)	11/9 29/24	25.61 (2.67) 28.2 (2.13)	MCI>HC MCI < HC	1 0	
	rs-fMRI	MCI 32 HC 53	73.99 (6.08) 76.08 (6.45)	17/15 29/24	22.9 (2.98) 28.2 (2.13)	MCI>HC MCI < HC	2 0	
Luo et al. (2018)	rs-fMRI	MCI 32 HC 49	72.43 (4.25) 73.33 (4.60)	17/15 18/31	27.16 (1.71) 29.02 (1.20)	CI>HC MCI < HC	1 1	*P < * 0.01 (cor)
		MCI 32 HC 49	74.90 (5.27) 73.33 (4.60)	17/15 18/31	28.34 (1.68) 29.02 (1.20)	MCI>HC MCI < HC	0 1	
Yuan X. et al. (2016)	rs-fMRI	MCI 36 HC 46	66.8 (9.5) 64.3 (7.8)	17/19 19/27	24.9 (3.4) 28.5 (2.0)	MCI>HC MCI < HC	4 3	*P < * 0.05 (uncor)
Wang et al. (2015)	rs-fMRI	MCI 30 HC 36	69.1 (5.8) 70.1 (5.5)	18/12 15/17	26.2 (2.2) 28.1 (1.5)	MCI>HC MCI < HC	3 3	
**fc**								
Liang et al. (2011)	rs-fMRI	MCI 16 HC 16	68.50 (7.77) 67.19 (8.38)	10/6 10/6	25.94 (1.65) 28.56 (0.63)	MCI>HC MCI < HC	2 1	*P < * 0.05 (cor)
Zhu et al. (2016)	rs-fMRI	MCI 19 HC 28	63.8 (6.7) 65.7 (10.7)	7/12 11/17	26.7 (1.6) 29.0 (0.8)	MCI>HC MCI < HC	0 2	*P < * 0.05 (cor)
Wu et al. (2014)	rs-fMRI	MCI 13 HC 16	69.00 (5.69) 67.75 (5.64)	6/7 8/8	26.23 (2.05) 29.13 (1.09)	MCI>HC MCI < HC	12 9	*P < * 0.05 (cor)
Yu et al. (2019)	rs-fMRI	MCI 14 HC 18	68.79 (8.99) 73.78 (9.92)	9/5 6/12	26.00 (0.88) 29.56 (0.51)	MCI>HC MCI < HC	1 1	*P < * 0.01 (uncor)
Liang et al. (2012)	rs-fMRI	MCI 14 HC 14	69.64 (6.88) 68.07 (7.46)	6/8 6/8	26.64 (1.01) 28.57 (0.65)	MCI>HC MCI < HC	1 4	*P < * 0.05 (cor)

### Meta-Analysis Results

#### Abnormal ALFF/fALFF in MCI

**Figure 2:**
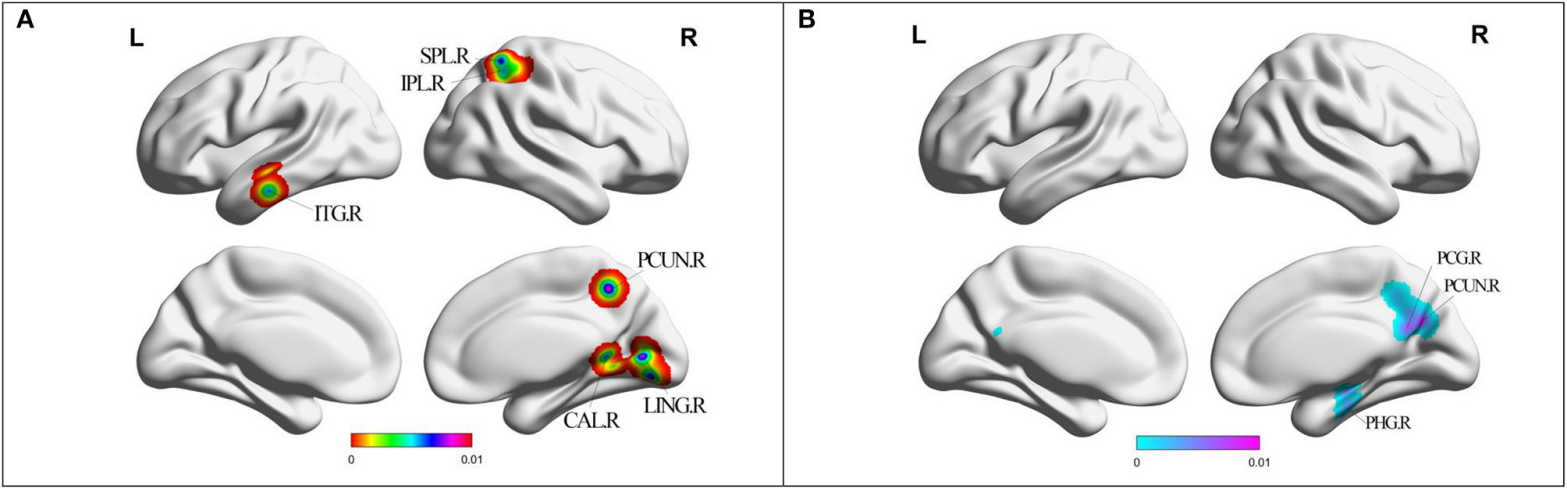
**(A)** Brain regions showing increased ALFF/fALFF in MCI patients compared with HCs. **(B)** Brain regions showing decreased ALFF/fALFF in MCI patients compared with HCs. MCI, amnestic mild cognitive impairment; HCs, healthy controls; ALFF/fALFF, the amplitude of low frequency fluctuation/fractional amplitude of low-frequency fluctuation; ITG, inferior temporal gyrus; CAL, cerebellum anterior lobe; LING, lingual gyrus; PCUN, precuneus; IPL, inferior parietal lobule; SPL, superior parietal lobule; PHG, parahippocampal gyrus; PCG, posterior cingulate; R, right; L, left.

Compared with HC, MCI patients showed increased ALFF/fALFF in the right lingual gyrus (LING), right cerebellum posterior lobe (CPL), right cerebellum anterior lobe (CAL), right precuneus (PCUN), right inferior parietal lobule (IPL), and left inferior temporal gyrus (ITG) (Table 2, Figure 2). MCI patients showed decreased ALFF/fALFF in right PCUN, right posterior cingulate cortex (PCC), and right parahippocampal gyrus (PHG) (Table 2, Figure 2).

**Table 2:** All clusters from ALE analysis.

**Cluster**	**Volume (mm^**3**^)**	**MNI**			**Anatomical regions**	**Maximum ALE value**	**Side**	**BA**
		**X**	**Y**	**Z**				
**ALFF/fALFF**								
**MCI>HC**								
1	25,160	12	−78	−6	Lingual gyrus	0.00939537	Right	18
1	25,160	10	−72	6	Lingual gyrus	0.008647516	Right	18
1	25,160	6	−64	−18	Cerebellum posterior lobe	0.009014993	Right	–
1	25,160	12	−52	4	Cerebellum anterior lobe	0.008706492	Right	–
2	21,880	34	−54	60	Superior parietal lobule	0.009608363	Right	7
2	21,880	30	−48	52	Precuneus	0.009242964	Right	7
2	21,880	10	−50	48	Precuneus	0.008747492	Right	7
2	21,880	50	−42	58	Inferior parietal lobule	0.0075041777	Right	40
2	21,880	42	−46	58	Inferior parietal lobule	0.0067537758	Right	40
3	12,680	−62	−18	−16	Inferior temporal gyrus	0.00849089	Left	20
3	12,680	−50	−18	−24	Sub-gyra	0.008587312	Left	21
**MCI < HC**								
1	12,080	12	−66	28	Precuneus	0.010766	Right	31
1	12,080	4	−58	24	Posterior cingulate	0.009437	Right	23
1	12,080	14	−52	44	Precuneus	0.006358	Right	7
2	9,720	36	−18	−16	Parahippocampal gyrus	0.008893	Right	–
2	9,720	24	−22	−32	Parahippocampal gyrus	0.00809	Right	35
2	9,720	20	−18	−20	Parahippocampal gyrus	0.008063	Right	28
**ReHo**								
**MCI>HC**								
1	21,424	56	14	14	Inferior frontal gyrus	0.008986	Right	44
1	21,424	54	18	0	Precentral gyrus	0.00663	Right	44
1	21,424	34	10	15	Claustrum	0.006546	Right	–
2	20,488	−18	−76	44	Precuneus	0.00918	Left	7
2	20,488	−26	−77	47	Precuneus	0.002658	Left	19
2	20,488	0	−70	36	Cuneus	0.006447	Left	7
3	18,920	9	−66	−6	Cerebellum anterior lobe	0.009139	Right	–
3	18,920	0	−90	−2	Lingual gyrus	0.008971	Left	18
3	18,920	14	−88	0	Lingual gyrus	0.00883	Right	18
4	15,392	−42	18	−9	Inferior frontal gyrus	0.008136	Left	47
4	15,392	−18	21	−9	Lentiform nucleus	0.00627	Left	–
5	13,208	16	−30	58	Paracentral lobule	0.008827	Right	5
5	13,208	20	−24	72	Precentral gyrus	0.00694	Right	4
**MCI < HC**								
1	18,512	−12	−48	34	Precuneus	0.009523	Left	31
1	18,512	−6	−48	34	Precuneus	0.007298	Left	31
1	18,512	0	−68	36	Cuneus	0.006447	Left	7
2	14,504	36	−72	54	Superior parietal lobule	0.009466	Right	7
2	14,504	44.7	−70	30	Middle temporal gyrus	0.004611	Right	39
**FUNCTIONAL CONNECTIVITY**								
**MCI>HC**								
1	14,984	46	30	32	Precentral gyrus	0.007362	Right	9
1	14,984	28	28	54	Middle frontal gyrus	0.007361	Right	6
1	14,984	36	42	38	Middle frontal gyrus	0.007333	Right	8
**MCI < HC**								
1	31,688	18	12	42	Cingulate gyrus	0.008292	Right	32
1	31,688	16	8	66	Medial frontal gyrus	0.007365	Right	6
1	31,688	−14	16	52	Medial frontal gyrus	0.007361	LEFT	6
1	31,688	−22	2	68	Superior frontal gyrus	0.007361	Left	6
1	31,688	4	6	58	Medial frontal gyrus	0.007126	Right	6
2	13,704	0	26	30	Cingulate gyrus	0.007361	Left	32
2	13,704	6	42	44	Superior frontal gyrus	0.007331	Right	8
3	13,256	36	30	21	Middle frontal gyrus	0.008029	Right	9
3	13,256	54	15	15	Inferior frontal gyrus	0.007415	Right	44

*BA, Brodmann Area; ALE, Anatomical/Activation Likelihood Estimation; MNI, Montreal Neurologic Institute; MCI, amnestic mild cognitive impairment; HCs, healthy controls; ALFF/fALFF, the amplitude of low frequency fluctuation/fractional amplitude of low-frequency fluctuation; ReHo, regional homogeneity; FC, functional connectivity*.

#### Altered ReHo in MCI

**Figure 3:**
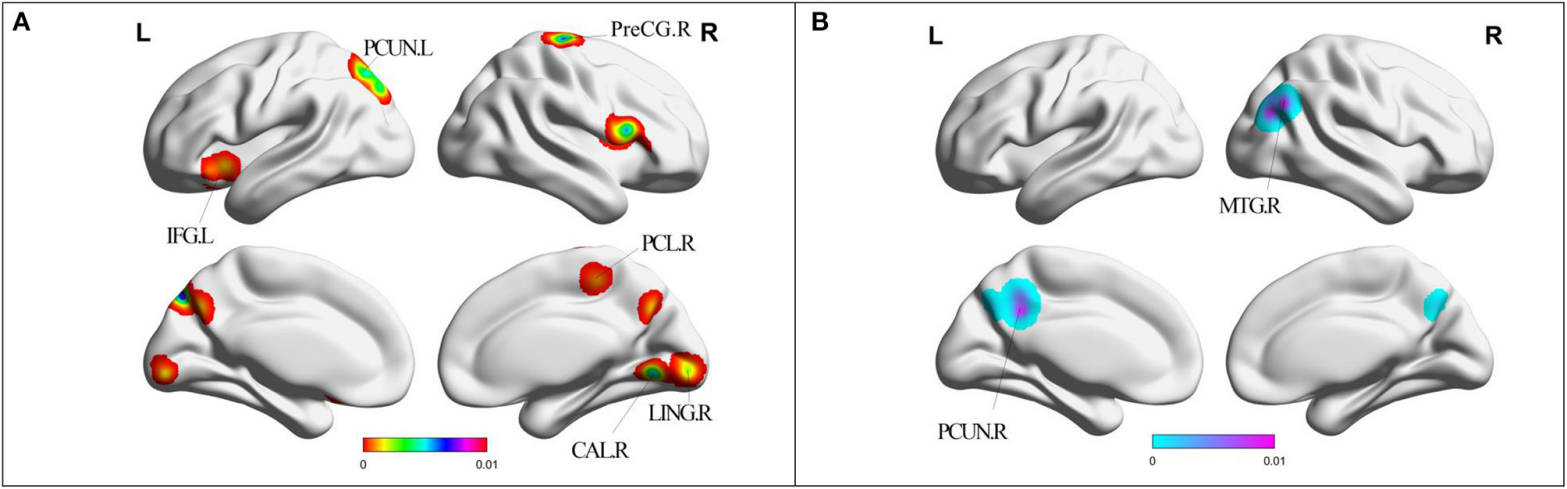
**(A)** Brain regions showing increased ReHo in MCI patients compared with HCs. **(B)** Brain regions showing decreased ReHo in MCI patients compared with HCs. MCI, amnestic mild cognitive impairment; ReHo, regional homogeneity; HCs, healthy controls; PHG, parahippocampal gyrus; PCG, posterior cingulate; PCUN, precuneus; MTG, middle temporal gyrus; LING, lingual gyrus; CAL, cerebellum anterior lobe; IFG, inferior frontal gyrus; PCL, paracentral lobule; PreCG, precentral gyrus; R, right; L, left. Cerebellum posterior lobe cannot be displayed in this template.

Compared with HC, MCI patients showed increased ReHo in left PCUN, right precentral gyrus (PreCG), left cuneus, bilateral LING, left inferior frontal gyrus (IFG), right paracentral lobule (PCL), and right PreCG (Table 2, Figure 3). MCI patients showed increased ReHo in bilateral PCUN, left cuneus, right superior parietal lobule (SPL), and right middle temporal gyrus (MTG) (Table 2, Figure 3).

#### Altered FC in MCI

**Figure 4:**
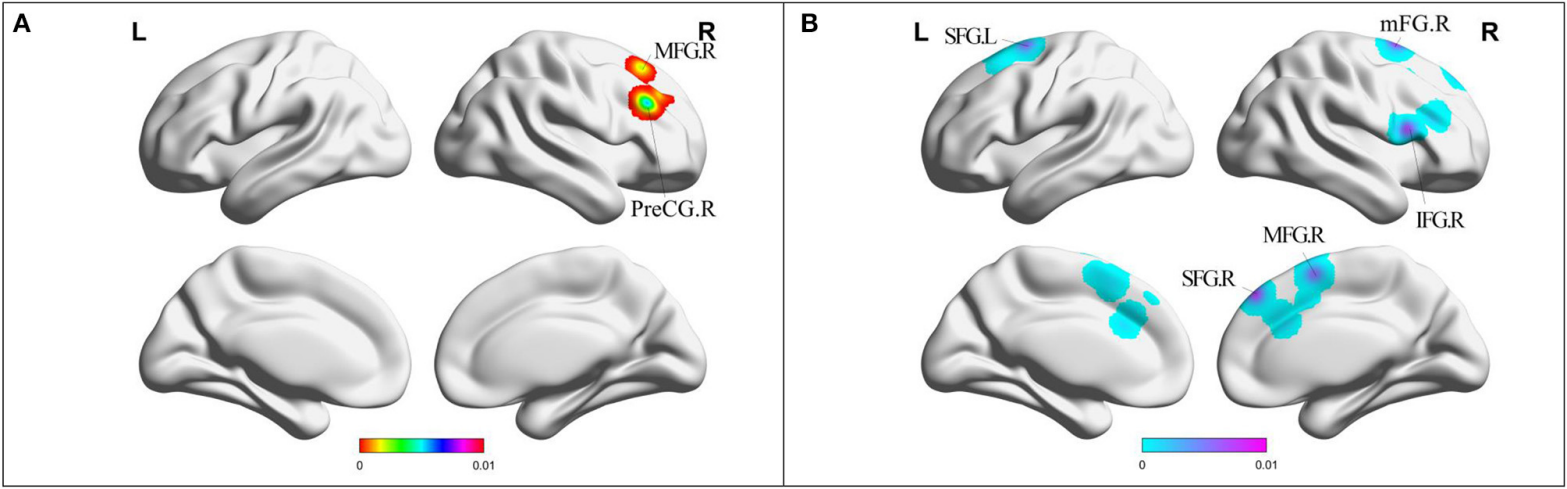
**(A)** Brain regions showing increased FC in MCI patients compared with HCs. **(B)** Brain regions showing decreased FC in MCI patients compared with HCs. MCI, amnestic mild cognitive impairment; FC, functional connectivity; HCs, healthy controls; PreCG, precentral gyrus; SFG, superior frontal gyrus; MFG, middle frontal gyrus; IFG, inferior frontal gyrus; mFG, medial frontal gyrus; R, right; L, left.

Compared with HC, MCI patients showed increased FC in right PreCG and right middle frontal gyrus (MFG) (Table 2, Figure 4). MCI patients showed decreased FC in right MFG, right IFG, bilateral cingulate gyrus, bilateral medial frontal gyrus (mFG), and bilateral superior frontal gyrus (SFG) (Table 2, Figure 4).

## Discussion

### Special Imaging Abnormal Markers

Our study was the first meta-analysis to identify specific brain region changes in the ECN and explore the interactions between the ECN and other networks. As expected, compared with HCs, the brain functional differences of MCI patients were observed in frontal regions, including mFG, IFG, SFG, and MFG, in addition to temporal regions, such as ITG, PHG, and MTG, as well as parietal regions, such as the PCC/PCUN, IPL, PCL, and SPL. There was little difference in the occipital lobes among the groups except for a small cluster of LING. Additionally, differences were also found in several subcortical regions, such as the lentiform nucleus (LN) and anterior and posterior lobe of the cerebellum.

In summary, reviewing the results of this paper, the ECN involved in frontal and parietal regions showed a downward trend in MCI patients. Areas of the ECN that showed an upward trend are mainly in the occipital, temporal, and subcortical regions with a small portion also rising in the frontal and parietal regions, which may be the compensatory mechanism of the disruption of frontal and parietal lobes in MCI patients (Valera-Bermejo et al., 2020). It is also well-established that the frontal and parietal regions are crucial aspects of the ECN, which send rich sensory information not only for movement controls, but also for other cognitive abilities, especially in executive function (Roh et al., 2020). Although the results reveal an increase in the frontal and parietal regions, these discrepant findings may be related to the different stages of MCI (Cosentino et al., 2020).

### Interactive Neural Network

The neural networks interacting with ECN involve DMN, VN, and FPN. The cerebro-cerebellar loops also were affected. Further analyses were performed to estimate whether the co-activation patterns of these regions could be fully attributed to some recognized neural networks.

The co-activation patterns, driven by differences between MCI and HCs, involved bilateral PCC/PCUN, right IPL, right PHG, and right MTG, which were mainly located in the DMN. The DMN is usually active when the individual is awake and resting, not focused on the outside world (Zhang et al., 2019). In contrast, when the individual is in the routine task experiment with external stimuli, the network was in the deactivated state (Zhang et al., 2019). Converging lines of evidence indicate that DMN, where β-amyloid (Aβ) protein deposition tends to occur, is considered to play an important role in various cognitive functions (Esposito et al., 2018). It is well-known that DMN contributes to information processing related to motivation, emotion, learning, cognition, and memory (Nicholson et al., 2020). In the present results, decreased ALFF/fALFF and ReHo in MCI were all jointly located in bilateral PCUN, which engage in executive function. In addition to the bilateral PCUN, the PCC, right PHG, and right MTG exhibit a significant decreased group difference. Recent research revealed that PCC and PCUN appeared to have the most structural and functional alterations in MCI patients (Fuchs et al., 2020). From the structural point of view, a study highlighted the association between PHG and gait parameters in MCI patients. There was a linear correlation between the decrease of hippocampal gray matter volume and the gait parameters representing executive function (Cosentino et al., 2020). The reason why most of the abnormal areas are in the right hemisphere is that adults' cognitive networks are biased (Katsel et al., 2018). However, the increased ALFF value in the PCUN and IPL, which may be evidence of a compensatory mechanism at different stages of disease (Simo et al., 2018). As reported, a compensatory mechanism in brain networks is common (Simo et al., 2018).

DMN is involved with episodic memory (EM) in which deficits are a typical clinical symptom of MCI (Yuan B. et al., 2016). Previous studies demonstrate that overlapping brain regions between the EF and EM networks indeed exist and elaborate that the DMN and ECN are dynamically interactive and closely linked (Yuan B. et al., 2016). Additionally, patients with EF impairment can also simultaneously suffer from impaired EM. It has been proven that dysfunctional connectivity in the DMN may reflect the gradual decline from MCI to AD (Hojjati et al., 2018). As a result, the present findings in the DMN show the neural mechanisms related to the interaction between impaired EF and the DMN network in MCI patients in new sights. Focusing on these brain areas is beneficial to the later individualized intervention for AD progression.

LING has the most significantly increased difference in terms of ALFF/fALFF and ReHo. Alteration of ALFF/fALFF also indicated an increase in ITG, and alteration of ReHo also indicated an increase in cuneus. However, decreased ReHo of cuneus was still revealed. If disturbed, LING, which is the important region of VN, fails to process visual function (Li et al., 2020). VN anatomy areas include LING, CUN, PCUN, middle occipital gyrus, and ITG (Li et al., 2020). Previous studies have elucidated that multiple integrations of SMN, VN, and the cognitive network coordinate with each other to provide the organism with the signals to perceive and respond to its surrounding environment (Xu et al., 2020). That is to say, impairments in any of these components will lead to cognitive impairment at the clinical level.

In the early stages of the disease, when defects occur in any of the DMN and ECN, the VN connection may increase as a compensation owing to the whole collaborative brain system. However, studies have shown that visual impairment does not necessarily occur when a pathway in the visual network is interrupted with the disease progressing (He et al., 2020). With multiple pathways impaired, patients may have problems with space, orientation, color, and size in visual areas, which further affect the executive function (He et al., 2020). Although our results show both a decrease and increase in cuneus, this suggests that cuneus is an area that is easily affected in cognitive impairment. Compensation and impairment are manifested at different stages of the disease (Simo et al., 2018).

Compared with HCs, MCI groups exhibit decreased functional connectivity in SFG, mFG, MFG, IFG, and cingulate gyrus. The area of decreased ALFF/fALFF in patients was as well as in cingulate gyrus. Increased key areas with ECN include MFG, SPL, PreCG, and IFG. These specific regions are located in the frontal-parietal cognitive/attention network (involved in many cognitive tasks, cognitive control, learning and planning, etc.) (Margolis et al., 2019). Based on resting-state functional connectivity and graph theory analysis, a two-system model for cognitive control, including FPN and the cingulo-opercular network, is proposed as the commanders of the brain (Cignetti et al., 2018). More specifically, the two networks that are hypothesized to be responsible for a dual-system of top-down control involve the cingulate cortex, dorsolateral prefrontal cortex (DLPFC), premotor cortex, anterior prefrontal cortex, IPL, medial frontal cortex, and superior portions of the parietal cortex (Cignetti et al., 2018).

A randomized controlled trial pointed out that executive functions, including control of thoughts and actions, fine-tuning attention, and information acquisition and analysis, are regarded to be involved in the attentional network model (van Houdt et al., 2019). As the important node of FPN, SPL is connected with visual attention. Our results show a functional disconnection within a distributed FPN network and confirm that disconnection of the prefrontal cortex and cingulate gyrus can also be detected in MCI, the early stage of AD. As we know, the frontal lobe is particularly advanced, involved in processing memory and emotion (Zhang et al., 2020). The anterior prefrontal cortex is a component gathered by IFG, SFG, MFG, and PreCG (Zhang et al., 2020). Current research confirms that the completion of executive function depends on the dynamic interaction between the prefrontal cortex and other cortical regions (Liang et al., 2012). In contrast, compensation coexists in MCI patients, which is the reason for the appearance of increasing frontal regions, especially in the early stage of AD.

In recent years, the role of the cerebellum in cognition has been gradually studied, especially in the collaborative management of cognitive functions within the brain. Posterior lobe lesions lead to cerebellar cognitive affective syndrome (CCAS), the typical characteristics of which involve deficits in executive function, linguistic learning, visual spatial processing, and regulation of affect (Wang et al., 2019). Previous studies demonstrate that the underlying mechanisms of cognitive impairment with cerebellar lesions might include abnormal fiber connections observed between the cerebellum and the functional areas of the cerebrum, particularly in the precuneus, cingulate gyrus, and frontal and temporal lobes (Schmahmann, 2019). In the present study, increased ALFF/fALFF in CPL and CAL were of certain important significance in the ECN. Considering the cerebro-cerebellar loops, it is reasonable to speculate that the increase of signal in the posterior cerebellum is a compensatory mechanism after the impairment of executive function in the MCI group (Schmahmann, 2019).

## Limitations

Although we have achieved valuable results, some shortcomings still need mentioning. First, the study is limited due to the existence of heterogeneity, including different data sources, preprocessing protocols, statistical or imaging methods, and threshold settings, which may have affected our results in this study. However, heterogeneity differences in study characteristics are negligible with respect to the changes in neuroimaging, which is a result from multicenter studies (Pardoe et al., 2008). Second, the subgroup analysis based on the MCI refinement classification cannot be carried out because the number of articles is inadequate. Third, confounding factors, such as age and gender, are as well as the unavoidable limitations.

## Clinical Implications

Although valuable clinical information is provided by a single study, it is essential to make quantitative neuroimaging analysis by summarizing the existing research results, which is helpful to consolidate our understanding of the pathophysiology of MCI. In terms of the significant overlapping regions, such as precuneus, cingulate gyrus, and lingual gyrus in ECN, it is no doubt that the findings of our article will provide new insight into cooperative interaction between ECN and other networks, which can be used as neural markers for disease improvement in different pathological states. TMS therapeutic target design and drug treatment will obtain a more objective basis. In a word, our results strongly reveal the imaging features of disease-specific brain area damage and lay the foundation for in-depth study of the pathogenesis of MCI.

## Conclusion

In the present study, we elucidate the evidence of special imaging biomarkers in the ECN and three abnormal neural networks whose core regions overlap in the ECN, including DMN, FPN network, cerebro-cerebellar loops, and interact with ECN with various methods of rsfMRI analysis. These findings provide evidence for further understanding the potential cerebral alterations in MCI. These meaningfully overlapping networks provide a new perspective for future selection of specific brain regions. However, further studies for classification of MCI among these networks are essential to explore the potential role of imaging as a biomarker for MCI.

## Author Contributions

WX guided by XL and JC designed the study. WX and SC performed the meta-analysis and drafted the manuscript. CX, GH, WM, and WQ helped in literature extraction and data analyses.

## Conflict of Interest

The authors declare that the research was conducted in the absence of any commercial or financial relationships that could be construed as a potential conflict of interest.
