# Supplementary material for: Functional MRI-Specific Alterations in Executive Control Network in Mild Cognitive Impairment: An ALE Meta-Analysis
Source: Front Aging Neurosci. 2020 Oct 9;12:578863. doi: 10.3389/fnagi.2020.578863 (PMC7581707; doi:10.3389/fnagi.2020.578863)
Supplement: Supplementary file 1 [file Table_1.DOC]

**Supplementary Table 1** Search terms for the systematic literature search

| **Database** | **Search terms** |
| --- | --- |
| PubMed | All Fields: 1) (“functional magnetic resonance imaging [MeSH] OR “RESTING STATE” [MeSH]) AND (“mild cognitive impairment”[MeSH] AND“Executive Control”[MeSH] AND “Functional connectivity”) ; 2) (“functional magnetic resonance imaging [MeSH] OR “RESTING STATE” [MeSH]) AND (“mild cognitive impairment”[MeSH] AND “regional homogeneity”); 3) (“functional magnetic resonance imaging [MeSH] OR “RESTING STATE” [MeSH]) AND (“mild cognitive impairment”[MeSH] AND (“fractional Amplitude of low frequency fluctuation” OR “amplitude of low frequency fluctuation”) |
| Web of Science | Same as Pubmed |
| Embase | Same as Pubmed |
